# Current Developments in Soil Ecotoxicology

**DOI:** 10.3390/toxics12100734

**Published:** 2024-10-11

**Authors:** Maria Luisa Fernández-Marcos

**Affiliations:** 1 Department of Soil Science and Agricultural Chemistry, Universidad de Santiago de Compostela, 27002 Lugo, Spain; mluisa.fernandez@usc.es; Tel.: +34-982823119; 2 Institute of Agricultural Biodiversity and Rural Development, Universidad de Santiago de Compostela, 27002 Lugo, Spain

Ecotoxicology focuses on how chemicals affect organisms in the environment, with the ultimate goal of safeguarding the structure and function of ecosystems [1]. Accordingly, soil ecotoxicology addresses the effects of toxic substances on soil ecosystems. Soil ecotoxicology is of paramount importance to soil health, with toxic substances being a major factor in the degradation of soil quality [2,3]. Recent research in this field has concentrated on the presence of various potentially toxic substances in soils, their mechanisms of action, and their ecological impacts. It has also explored methods for assessing the ecotoxicology of pollutants and facilitated the development of sensitive and precise analytical techniques for detecting and quantifying contaminants in soil environments. There has been significant advancement in this area with the recent development of models that predict the effects of contaminants on soil health. Additionally, numerous studies have focused on strategies for the remediation and mitigation of contaminated soils.

Metals and other trace elements [4,5], pesticides [6,7,8], polycyclic aromatic hydrocarbons (PAHs) [9,10,11], antibiotics and other pharmaceuticals [12,13] are particularly harmful to soil ecosystems. Emerging contaminants, such as rare earth elements [14], microplastics and nanoplastics [15,16,17,18], and engineered nanomaterials [19,20,21,22], are also in need of attention. These substances can enter the soil through various pathways, including direct application (e.g., fertilizers, pesticides), atmospheric deposition (e.g., heavy metals, PAHs), irrigation with contaminated water, the use of biosolids and sewage sludge in agricultural soils, industrial spills, and the degradation of waste products such as plastics.

Assessing soil ecotoxicology involves a variety of methods aimed at evaluating both the presence of contaminants and their potential ecological impacts. These methods can be broadly classified as chemical analysis, bioassays, molecular techniques, and ecological modeling. Common bioindicators include earthworms [23,24,25], plants [5], and soil-dwelling arthropods [26,27,28]. Advances in molecular biology have enabled techniques that assess the impact of pollutants on soil microbial communities at the genetic level, which may disrupt essential soil processes such as nutrient cycling and organic matter decomposition [29,30,31].

This Special Issue comprises several articles that address various aspects of soil ecotoxicology.

Three articles investigated the presence and effects of potentially toxic trace elements in soils. Enamorado-Montes et al. (Contribution 1) examined mercury accumulation in three varieties of *Oryza sativa* L. grown in Hg-contaminated soils and assessed its impact on rice yield and quality. They observed no phytotoxic damage at concentrations up to 1.5 mg Hg per kg of soil. Their results indicated that mercury accumulation was highest in the roots, followed by the husks and grains. The potential risk to human health posed by rice consumption was evaluated using the United States Environmental Protection Agency (USEPA) methodology, and there was noticeable variability among rice varieties and mercury soil concentrations.

Santos et al. (Contribution 2) investigated the behavior of chromium in soil systems. The soil annelid species *Enchytraeus crypticus* was used to assess the toxicokinetics of Cr(III) in soil. Exposure to chromium resulted in rapid uptake and elimination. When the annelids were transferred to uncontaminated soil, their chromium levels returned to baseline within approximately seven days. This study highlights the critical role of toxicokinetic studies in assessing toxicity.

Cepoi et al. (Contribution 3) address the presence and removal of copper and other metal ions in soil systems. Their article focuses on the bioremediation of these soils using the cyanobacterium *Nostoc linckia*, which is known for its ability to accumulate metal ions. The accumulation of Cu, Fe, Ni, and Zn in the biomass was measured through neutron activation analysis. *N. linckia* demonstrated a strong capacity to accumulate copper and other metal ions from multi-elemental systems.

The article by Jorge-Escudero et al. (Contribution 4) examines the impact of two commercial fungicides on earthworms, emphasizing the potential interactions among active ingredients and the significance of other ingredients alongside the active ingredient. The fungicides tested were SWINGPLUS (BASF^®^), which contains metconazole and epoxiconazole, and PROSARO (BAYER^®^), which includes tebuconazole and prothioconazole as active ingredients. Both are recommended for controlling fusarium head blight in wheat. *Eisenia fetida* and *Glossoscolex rione* were used as test organisms. In an experiment investigating sublethal effects on *E. fetida*, both fungicides were applied at five different concentrations: one that was an order of magnitude lower than the dosage recommended by commercial manufacturers for field use; the recommended concentration; and three concentrations that were one, two, and three orders of magnitude higher than recommended. In a second experiment testing lethality in both *E. fetida* and *G. rione*, PROSARO was applied at five concentrations following a logarithmic series, spanning the range between the two highest concentrations from the first experiment. The study indicates that these commercial fungicides’ toxicity to earthworms may be significantly higher than the sum of the toxicities of their individual active ingredients. Moreover, the results suggest a higher sensitivity of *G rione* to the fungicide PROSARO compared to the standard test organism, *E. fetida*, indicating a possible underestimation of agrochemicals’ toxicity when using *E. fetida*.

Mendes et al. (Contribution 5) investigate the effects of contaminant mixtures by examining the combined effects of nanopolystyrene and other contaminant substances (diphenhydramine, which is representative of pharmaceuticals; silver nitrate, which is representative of metals; and vanadium nanoparticles, which are representative of nanomaterials) on the soil invertebrate *Enchytraeus crypticus*. Their study shows that combining diphenhydramine at 10 and 50 mg/kg with 300 mg/kg of nanopolystyrene leads to a reduction in reproduction, demonstrating a significant interaction between these contaminants (synergism). The results indicate that nanoplastics may act as carriers for other contaminants and potentiate the effects of pharmaceuticals, highlighting the detrimental impact of contaminant mixtures on soil ecosystems.

Landi et al. (Contribution 6) employ a combination of proteomics and a bioassay to assess contaminants in a topsoil sample from an industrial area and evaluate the associated risks to human and environmental health. Their innovative approach, which couples proteomics with a bioassay, successfully detects PCDD/Fs, dioxin-like compounds, and PAHs, confirmed by GC-MS/MS analysis. Additionally, the article suggests that combining effect-based tools with proteomics could offer a promising new approach to the risk assessment and monitoring of complex matrices, such as soils. The authors recommend additional validation campaigns to strengthen the reliability of the proposed approach for assessing the presence of complex mixtures, including pharmaceuticals, and their effects on living organisms, including humans, based on soil use.

Barron and Lambert’s article (Contribution 7) focuses on modeling soil ecotoxicity. They develop interspecies correlation estimation (ICE) models for soil invertebrates, which enable the prediction of toxicity to one species based on known toxicity to another. Utilizing data from 12 soil invertebrate species and 192 chemicals, they develop ICE models for 11 species pairs. The results demonstrate high prediction accuracy within the same order (e.g., earthworm to earthworm), but lower accuracy for models predicting toxicity across different taxa (e.g., Arthropoda to Annelida and vice versa).

Camacho et al. (Contribution 8) investigate how different land uses affect soil quality and microbial diversity. They examine three land cover types—undisturbed forests, degraded shrublands, and non-irrigated croplands—on poorly developed Mediterranean soils in Spain. Based on gene sequencing analyses of microbial communities of Bacteria, Archaea, and ectomycorrhizal fungi, they determine that forest soils have greater soil organic matter, higher microbial richness and biomass, and more stable and interconnected ecological networks. These findings highlight the potential risks that deforestation and agricultural practices pose to the conservation of soil microbiota.

The review featured in this Special Issue (Contribution 9) explores the impact of wildfires on soil ecotoxicology. Wildfires not only alter various chemical, physical, and biological properties of soil but also generate a complex mix of potentially harmful substances that can attain (and thus damage) soil and water bodies. This review aims to summarize the current understanding of how wildfires produce toxic substances, their effects on soil organisms, and the associated risks. Numerous studies have documented the inhibitory effects of ash on seed germination and seedling growth, as well as its toxicity to soil and aquatic life. There is a wealth of research on the mobilization of heavy metals and trace elements by fire, as well as the presence of polycyclic aromatic hydrocarbons (PAHs) and other organic pollutants in post-fire soils, along with their toxicity to plants, soil, and aquatic organisms. The review also considers the potential role of charcoal in detoxifying fire-affected soils.

The articles included in this publication cover various aspects of soil ecotoxicology and identify various areas in need of more research. The most notable under-addressed topics are as follows:

- The toxicokinetics of chromium and other potentially toxic elements in soil organisms. Assessing toxicity over time is a crucial method for evaluating the risks posed by metals (Contribution 2).
- Metal bioaccumulation in monometallic systems, further studying the metal accumulation capabilities of *Nostoc linckia* biomass (Contribution 3).
- The interactions and toxicity mechanisms of nanoplastics, particularly when they are part of complex contaminant mixtures. A key area of study should be the molecular interactions between nanoplastics and other components, such as natural organic matter or other pollutant matrices like wastewater sludge (Contribution 5).
- These studies have highlighted the need for additional toxicity data for soil invertebrates, obtained through single chemical tests in soil media. The existing data are largely limited to earthworms, while other taxa remain underexplored (Contribution 7).
- Beyond using the standard test organism *Eisenia fetida* as a bioindicator of soil contamination, it is necessary to conduct further research on other earthworm species commonly found in agricultural fields or native species, such as *Glossoscolex rione*. Additionally, it is important to assess the toxicity of commercial products, not just the active ingredients in pesticides (Contribution 4).
- A detailed study of soil microbial composition across a degradation gradient is needed, and should examine how microbial communities change in response to soil degradation due to different land uses. Such research would inform the selection of land use management practices to improve soil quality (Contribution 8).
- Regarding the review on potentially toxic substances in soils affected by wildfires (Contribution 9), several research gaps have been identified:
  - The vertical and lateral distribution of toxic substances generated by fires.
  - There is a need for more studies that integrate ecotoxicological research on burnt soils with the identification and quantification of the substances that cause the toxicity, rather than focusing on just one of these aspects.
  - PAHs analysis should be adapted to reflect emerging criteria for selecting individual PAHs indicative of environmental or health hazards. Additionally, there is a need for further analysis of other organic pollutants, such as PCBs and dioxins, as very little research has been conducted in this area thus far
